# An Assessment of Risk Factors for Insufficient Levels of Vitamin D during Early Infancy

**DOI:** 10.3390/nu13041068

**Published:** 2021-03-25

**Authors:** Keith T. S. Tung, Rosa S. Wong, Hing Wai Tsang, Bianca N. K. Chan, Siew Yan Wong, Hung-Kwan So, Joanna Y. L. Tung, Marco H. K. Ho, Wilfred H. S. Wong, Patrick Ip

**Affiliations:** 1 Department of Paediatrics and Adolescent Medicine, The University of Hong Kong, Hong Kong SAR 000000, China; keith-tung@connect.hku.hk (K.T.S.T.); rosawg@connect.hku.hk (R.S.W.); thwpaed@hku.hk (H.W.T.); bbianca@hku.hk (B.N.K.C.); yanws@hku.hk (S.Y.W.); hkso@hku.hk (H.-K.S.); tungylj@hku.hk (J.Y.L.T.); marcoho@hku.hk (M.H.K.H.); whswong@hku.hk (W.H.S.W.); 2 Department of Paediatrics, Hong Kong Children’s Hospital, Hong Kong 000000 SAR, China

**Keywords:** serum 25-hydroxyvitamin d, vitamin d, infants, breastfeeding, epidemiology, cumulative risk model

## Abstract

Recent evidence suggests that breastfeeding may increase the risk of vitamin D deficiency in offspring. However, it is unclear whether increased risk results from breastfeeding alone, or whether it is associated together with other risk factors. This study surveyed 208 infant–mother dyads recruited by stratified random sampling in different districts of Hong Kong. Mothers were asked to complete a questionnaire on their demographics, history of risk behavior, and feeding practices. Peripheral blood samples were collected from infants to determine their vitamin D status. Among all infant participants, 70 were vitamin D insufficient or deficient. Being breastfed, being a girl, having a multiparous mother, and the use of sun cream were found to be the strongest risk factors for vitamin D insufficiency during infancy (all *p* < 0.05), after mutual adjustment. The cumulative risk model displayed a dose–response pattern between the number of risk factors and the risk of vitamin D insufficiency during this period. Our findings indicate the risk profile of infants with insufficient vitamin D. Guidelines and recommendations on healthy diet and lifestyle should be provided to mothers during the early stage of pregnancy to increase the likelihood of adequate levels of vitamin D in their offspring.

## 1. Introduction

Breastfeeding is considered to be the most preferred and nutritious food source for infants. The World Health Organization recommends that infants in their first six months of life should be breastfed exclusively. An increasing trend in breastfeeding practices in Hong Kong has been reported, after persistent efforts to publicize the benefits of breastfeeding through large-scale campaigns [1]. However, breast milk is intrinsically limited by a relatively low vitamin D content. A previous review found that the mean vitamin D concentration in the breast milk (as expressed by antirachitic activity) of healthy lactating women, irrespective of whether they were supplemented, should range from 10 to 80 IU/L [2]. The concentration of vitamin D in breast milk can be influenced by various factors, such as amount of sun exposure, supplementation practices, and ethnicity of lactating mothers [2,3,4]. Exclusively breastfed infants are often unable to meet their daily vitamin D requirement without supplementation or adequate sunlight exposure [5,6], and, thus, they represent the most vulnerable group at risk of vitamin D insufficiency. Furthermore, even though breastfed infants are recommended to take supplements up to the age of two years or beyond, until solid foods are introduced [7], parental non-compliance and inconsistent guidelines from practitioners could limit the efficiency and effectiveness of vitamin D supplementation [8,9], putting breastfed infants at a higher risk of vitamin D deficiency.

Vitamin D deficiency among infants and toddlers is a public health problem worldwide. The reported prevalence of vitamin D deficiency in exclusively breastfed infants ranges from 27% to 82% [2], with the highest rate reported in Asian populations [10]. High prevalence of vitamin D deficiency warrants attention because of the key role of vitamin D in calcium absorption, bone mineralization, and immunomodulatory functions [11]. Children suffering from chronic vitamin D deficiency often develop growth retardation, skeletal deformities, impaired immune systems, and respiratory infections during early childhood. [12]. Findings from previous studies also suggest that vitamin D deficiency is a risk factor for many chronic diseases, including type 1 diabetes mellitus, multiple sclerosis, and cardiovascular disease [13,14,15]. Hence, maintaining vitamin D at a sufficient level from childhood would have implications for the prevention of osteoporosis and other chronic diseases later in life.

However, vitamin D levels vary widely between individuals due to individual differences in environmental exposures and endogenous factors. It is particularly challenging to achieve adequate vitamin D levels in early life because of limited sunlight exposure and dietary options [16]. Although previous studies reported that factors such as low socioeconomic class and lack of supplementation could increase the risk of vitamin D deficiency [17,18], these factors rarely operate in isolation but, instead, often occur simultaneously. Cumulative exposure to multiple risk factors at one point in time, or over time, rather than exposure to a single factor, may have a stronger impact on health and health-related conditions, including vitamin D status [19]. While this cumulative risk analysis approach has been frequently used to examine the effect of environmental risks on pediatric health outcomes [20], evidence using such a model to explain vitamin D levels, particularly during infancy, remains very limited. 

Against this background, this study has two aims. First, it examined the associations of individual factors, including feeding practices, sun exposure, supplementation patterns, and demographic characteristics, with the risk of vitamin D insufficiency among those exclusively and partially breastfed infants, respectively. Second, we applied the cumulative risk analysis method to study the effects of breastfeeding practice, alone and together with other risk factors, on the level of vitamin D in these infants. Specifically, we hypothesized that the risk of vitamin D insufficiency would be higher in breastfed infants with more risk factors.

## 2. Materials and Methods

### 2.1. Study Design and Participants

This was a population-based, cross-sectional study exploring the risk factors for vitamin D insufficiency using data of infants aged 2 to 6 months recruited by stratified random sampling in different districts of Hong Kong. Infants with any major congenital malformations, or those born premature or with low birth weight, were excluded from this study. During the period of July 2019 to November 2020, a total of 208 infant–mother dyads who visited the maternal and child health centers were approached and provided informed consent to participate in this study. Upon informed consent, the mothers were asked to complete a questionnaire on their demographics, history of risk behavior and feeding practices. Peripheral blood samples were collected from the infants by a well-trained phlebotomist. An incentive of HKD200 (approximately USD 25.6) supermarket voucher was given to the participants upon the completion of the study assessment. 

### 2.2. Measures

The following describes our determination of the infants’ vitamin D status. Serum extracted from the collected peripheral blood samples was used to determine the infants’ vitamin D status measured by the total serum 25-hydroxyvitamin D (25(OH)D) concentration. With high specificity and precision showed in previous studies [21], a liquid chromatography–tandem mass spectrometry method was adopted in this study to determine the infants’ vitamin D status. The AB Sciex QTRAP 5500 LC-MS/MS system was used to simultaneously detect the concentration of 25(OH)D3, 25(OH)D2, and 3-Epi-25(OH)D3. Our LC-MS/MS method was shown to be linear using a 6-point calibration curve with an R^2^ value over 0.99. Calibration of the LC-MS/MS method was also verified against the samples from the Vitamin D External Quality Assessment Scheme, in which our results were within ±15% of the target value, for a total of 25(OH)D [22]. The total serum 25(OH)D concentration, defined as the sum of 25(OH)D3 and 25(OH)D2 minus by 3-Epi-25(OH)D3, of less than 50 nmol/L and 25 nmol/L, are the cut-off points for vitamin D insufficiency and deficiency, respectively. The cut-off used in this study was recommended in the clinical practice guidelines of the Endocrine Society Task Force on Vitamin D [4,23]. In this study, serum 25(OH)D concentration of less than 50 nmol/L was used to dichotomize the infants into the vitamin D sufficiency and insufficiency (including those with vitamin D deficiency) groups. 

The following describes our analysis on feeding practice. An Infant Feeding Category Assessment Tool (FeedCat Tool) was completed by the mothers to describe infant feeding patterns, including the duration of breastfeeding and the degree of breastfeeding exclusivity. The FeedCat Tool was designed to have two sections, with the first part containing questions on what and how the baby was fed, and the second part having a chart to indicate the feeding category for the infants. The FeedCat Tool has been demonstrated by previous studies to have good validity and reliability [24]. Infants who were ever breastfed, both partially and exclusively, in the past seven days were categorized as breastfed infants. Mothers were also asked to report whether they provided any vitamin D supplements to their infant, such as fish liver oil and multivitamins. 

What follows is our determination of the infants’ sun exposure. Items adopted from the diet and nutrition survey of infants and young children were used to reflect the sun exposure of the infants [25]. Mothers were asked to report whether they regularly apply any sun cream to their infant, which was used as a proxy to estimate the infant’s level of sun exposure in this study. The mean ultraviolet (UV) indices at the date of the visit of the participants were obtained from the Hong Kong Observatory. The UV index is a measure of exposure level to UV radiation on a scale of 0 (Low exposure) to 10+ (Extreme exposure) [26,27].

Maternal smoking history was a binary variable (with or without smoking history) in this study, and was based on the mother’s self-report of whether they had ever smoked.

The following describes our analysis of demographic characteristics for this study. Maternal and child characteristics, including the age and sex of the infants, as well as the educational level, employment status, current marital status, and parity of the mothers, were obtained. In addition, the mothers were asked to report their family monthly income level as an indicator of their socioeconomic status. In this study, the mothers were categorized into three income groups: low income (less than HKD $29999), median income ($30000 to $39999), and high income (higher than $39999).

### 2.3. Data Analysis 

Descriptive statistics were used to summarize the demographic and family characteristics of the infant–mother dyads. Between-group comparisons were made by a series of independent *t*-tests (for continuous variables) and chi-square analyses (for categorical variables) to determine differences in family and infant characteristics, and maternal behaviors between sufficient and insufficient vitamin D groups. 

A series of logistic regression models were then fitted to examine the relationships between different factors and vitamin D insufficiency. An adjusted model was constructed for each individual-level risk factor, with age and sex of the infants and the mean UV index at month of interview included as covariates. All factors were then put into the same model for mutual adjustment to identify the predominant risk factor for vitamin D insufficiency. Multicollinearity among variables was also checked, with no indication found. 

To examine the effect of cumulative risk factors on vitamin D status, infants were categorized into five groups: a) non-breastfed, b) breastfed with 1 risk factor, c) breastfed with 2 risk factors, d) breastfed with 3 risk factors, and e) breastfed with 4 or more risk factors. Adjusted regression analyses were first performed to understand the risk of vitamin D insufficiency among breastfed infants with different numbers of risk factors. The same analyses were conducted for both exclusively and partially breastfed infants. Statistical significance was determined at *p* < 0.05 by two-tailed tests. All analyses were conducted using SPSS Statistics (version 26.0, IBM Corp, Armonk, NY, USA).

## 3. Results

Table 1 displays the sociodemographic characteristics of the 208 mother–infant dyads. The overall sample involved 112 boys (53.8%) and 96 girls (46.2%), with an average age of 4.36 months (Standard deviation (SD) = 1.6). Among the 208 infants, 76 (36.5%) were partially breastfed and 66 (31.7%) were exclusively breastfed. In this study, 77 infants were vitamin D insufficient, of which 31 were vitamin D deficient. The mean serum 25(OH)D level among all infants was 59.5 nmol/L (Range: 2.7 to 169.9 nmol/L). Compared to the sufficient vitamin D group, the vitamin D insufficiency group was more likely to be younger, fed by breastmilk, and have multiparous mothers.

Table 2 displays the results of the regression analyses on various factors that could be associated with vitamin D insufficiency in infants. After adjusting for the age and sex of the infants and the mean UV index at the month of interview, having a multiparous mother (aOR = 4.04, *p* = 0.002), the application of sun cream by parents (aOR = 4.08, *p* = 0.017), and being breastfed (aOR = 12.81, *p* < 0.001), regardless whether exclusively or partially, were significantly associated with a higher risk of vitamin D insufficiency. Girls were also found to have a higher risk of vitamin D insufficiency than boys (aOR = 1.87, *p* = 0.036), after being adjusted for the infant’s age. The associations remained significant after mutual adjustment. 

**Figure 1:**
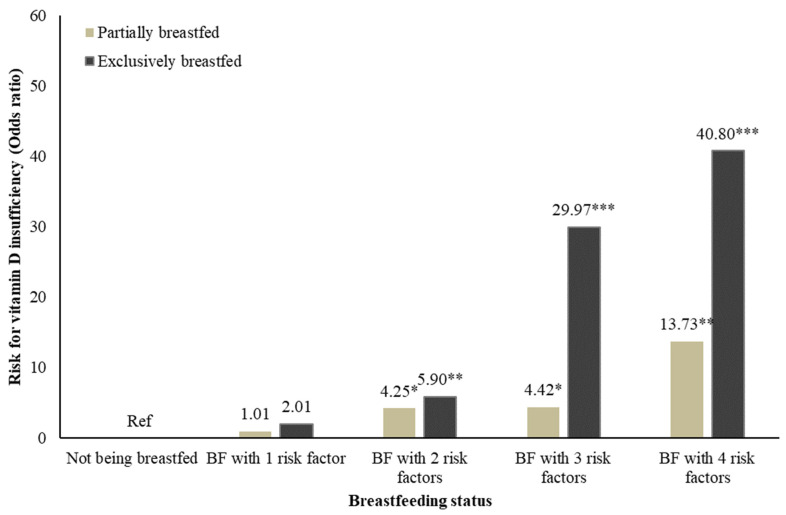
Cumulative risk model on vitamin D insufficiency or deficiency risk among breastfed infants. Note: * *p* < 0.05, ** *p* < 0.01, *** *p* < 0.001, BF: Breastfed, EBF: Exclusively breastfed.

As shown in Figure 1, our study found a “piling up” effect of risk exposures on vitamin D insufficiency risk during infancy. Compared to non-breastfed infants, infants who were breastfed and exposed to multiple risks had a significantly higher risk of vitamin D insufficiency (aOR for 2 risk factors: 4.25 to 5.90 3 risk factors: 4.42 to 29.97, aOR for 4 risk factors: 13.73 to 40.80, all *p* < 0.01). However, the risk was not elevated when there was only one risk factor in the breastfed infants.

## 4. Discussion

This study examined the independent and cumulative effects of different risk factors on vitamin D status among infants aged 2 to 6 months in Hong Kong. Our results showed that being a girl, having a multiparous mother, having sun cream applied by parents, and being breastfed were the strongest risk factors for vitamin D insufficiency during early infancy. Furthermore, our cumulative risk analysis found a “piling up” effect of cumulative risk exposures in predicting the risk of vitamin D insufficiency among infants. Specifically, there was an eight-fold increase in the risk of vitamin D insufficiency among breastfed infants exposed to four risk factors when compared to those exposed only to two risk factors; there was even a twenty-fold increase when compared to those exposed to only one risk factor. Our results add to the literature by showing that breastfed and non-breastfed infants are equally likely to suffer from insufficient vitamin D, in the absence of other factors that may influence the effect of breastfeeding practices on the offspring’s 25(OH)D concentration. 

These findings partially echo previous evidence on the increased risk of vitamin D deficiency among breastfed infants [28]. This is because, by using the cumulative risk analysis approach, we found that the occurrence of multiple risk factors is the main reason for higher risk of vitamin D insufficiency or deficiency among breastfed infants. With local and international efforts to promote breastfeeding, breastfeeding has become a popular feeding practice, especially during early infancy. However, breastfed infants may not meet the recommended level of vitamin D intake of 400IU per day due to the relatively low vitamin D concentration in breastmilk (10 to 80 IU/L) [29,30,31]. Indeed, breastfed infants were often found to be vitamin D deficient [12,32]. Consistent with these previous findings, our study also found that over half of the breastfed infants were vitamin D insufficient or deficient. Furthermore, our study demonstrated the presence of other factors that could potentially increase the risk of vitamin D insufficiency or deficiency during early infancy. Together, these findings indicate the likelihood of cumulative risk exposure as a potential cause of vitamin D deficiency among breastfed infants. 

In addition, our results highlight the vulnerability of breastfed infants, aged 2 to 6 months, to vitamin D insufficiency, especially when they are simultaneously exposed to multiple risk factors. During their first six months, infants generally obtain vitamin D from the transplacental store, dietary intake, and cutaneous production via sunlight [33]. It has been reported that vitamin D is reserved in the body of an infant at birth through a trans-placental passage from the mother during the intrauterine period [34]. However, their vitamin D reserves would eventually be exhausted if infants are unable to meet the recommended daily intake amount due to limited dietary intake and sunlight exposure [9,35]. Without vitamin D supplementation, infants in this early period of life could be more vulnerable to vitamin D insufficiency or deficiency. 

To gain a more comprehensive understanding of what constitutes the risk of vitamin D deficiency in breastfed infants, we conducted a cumulative risk analysis with various risk factors, as reported in previous studies; these risk factors include: the infant being a girl, a low family income, having a multiparous mother, no vitamin supplementation, sun cream being applied by their parents, and a mother with smoking history. Since breastmilk cannot provide adequate vitamin D to infants, vitamin D supplementation and cutaneous synthesis are key alternative sources for infants to achieve the daily recommended intake amount [9]. In addition, compared to disadvantaged families, higher income families were more able to provide a healthy diet, including more supplementation and various nutrients to their children [36,37,38]. Cigarette smoke is another commonly reported factor that could negatively affect vitamin D levels, as the smoke can decrease the production of active forms of vitamin D and tamper with the expression of vitamin D receptors [39]. It is proposed that reduced vitamin D content in mothers with a smoking history may lead to an increased risk of vitamin D insufficiency or deficiency in their infants [40]. While these factors were not found as the strongest risk factors in this study, they constituted the cumulative risk profile for breastfed infants with vitamin D insufficiency.

In this study, being a girl, sun cream being applied by parents, and having a multiparous mother were the strongest risk factors for vitamin D insufficiency during early infancy. Being consistent with previous evidence [41], our results showed that infant girls were more likely than boys to be vitamin D insufficient or deficient. One possible reason for such a difference in sex could be that parents have a higher tendency to apply sun cream to girls. Based on recent evidence from vitamin D related genotyping studies [42], gene-by-sex interaction may exist to affect infants’ susceptibility to vitamin D deficiency, although further research is needed to substantiate this possibility. Given there is no clear evidence on the potential of gene-by-sex interaction, the presenting of gender differences in the risk of vitamin D insufficiency may not be due to the gender itself, but, instead, could be influenced by other related factors. Given the international guidelines for infants under 6 months of age to stay away from direct sun exposure [43], some parents may limit sunlight exposure for their infants. Some may even apply sun cream on their infants for sunlight protection, which could further reduce vitamin D synthesis, up to 95%, through skin [44]. Meanwhile, infants born to multiparous mothers were also more likely than those born to uniparous mothers to have vitamin D insufficiency or deficiency. Previous research suggested that, compared to uniparous mothers, multiparous mothers tend to breastfeed their children for a longer period because of previous breastfeeding experience [45,46]; and yet, they are also in poorer health, which might further reduce the vitamin D content in their breastmilk [47]. Moreover, uniparous mothers were found to take better care of their first child [48]. All these factors may account for the higher risk of vitamin D insufficiency or deficiency among infants born to multiparous mothers.

This study has several limitations. First, this was a cross-sectional study and, therefore, we were not able to establish a causal relationship between different risk factors and vitamin D status. Second, because of our relatively small sample size, this study cannot generalize findings to the whole population. However, we attempted to enhance the study’s representativeness by conducting recruitment at multiple centers across the territory. Third, we did not take the potential dietary transition to solid-based food intake by infants into consideration, which might potentially bias our results. As previous research has demonstrated the potential differences in vitamin D content between liquid- and solid-based food [9], future research would benefit from including this information. Nevertheless, the percentage of infants who begin to have a solid-based food intake by 6 months of age should be minimal. Fourth, our cut-off value of vitamin D insufficiency (50nmol/L) was selected based on previous data for bone health; the ideal level for other outcomes has yet to be determined. Lastly, our cumulative risk model did not include other potential risk factors, such as maternal vitamin D level and nutritional knowledge. Further research should examine a more comprehensive risk factor profile specific for vitamin D insufficiency in breastfed infants.

## 5. Conclusions

In conclusion, the results of this study support our hypothesis that the risk of vitamin D insufficiency could be higher among breastfed infants who experience more risk factors. Vitamin D is essential for infants’ growth, bone mineralization, immunomodulation, and cell regulation [11]. The role of vitamin D is particularly important during infancy and early childhood, as it can foster the development of cognitive, motor and socio-emotional skills during this period [49,50]. Chronic vitamin D insufficiency, on the other hand, can lead to rickets and other long-term impairments in growth and intellectual performance [51,52]. Therefore, it is important to maintain vitamin D at a sufficient level during early infancy to optimize growth and development. The results of our cumulative risk analysis add to the literature by highlighting the effects of the interaction between breastfeeding and other risk factors that could potentially increase the risk of vitamin D insufficiency, or even deficiency, among breastfed infants. Providing adequate vitamin D supplementation and risk factor assessment is thus beneficial, particularly for exclusively breastfeeding infants. Clinicians should not only promote breastfeeding but also have the ability to identify vulnerable groups at risk of vitamin D insufficiency early. Adequate support and guidance, such as the provision of supplementation and information on the importance of sufficient vitamin D intake, could then be provided to mothers of these high-risk infants to minimize their subsequent risk of vitamin D insufficiency. 

## Figures and Tables

**Table 1:** Demographics of the participants

	Total	Sufficient Vitamin D (25(OH)D > 50 nmol/L)	Insufficient Vitamin D (25(OH)D ≤ 50 nmol/L)	*p*-Value
	(*N* = 208)	(*N* = 131)	(*N* = 77)	
Age, month, mean (SD)	4.36 (1.6)	4.69 (1.4)	3.79 (1.6)	<0.001
Sex, *N* (%)				0.084
Boys	112 (53.8%)	77 (58.8%)	35 (45.5%)	
Girls	96 (46.2%)	54 (41.2%)	42 (54.5%)	
Mother’s occupation, *N* (%)				0.462
Housewife/ Home maker	91 (43.8%)	60 (45.8%)	31 (40.3%)	
Full-time employed	100 (48.1%)	58 (44.3%)	42 (54.5%)	
Part-time employed	6 (2.9%)	5 (3.8%)	1 (1.3%)	
Mother’s educational level, *N* (%)				0.117
Lower secondary education or below	19 (9.1%)	12 (9.2%)	7 (9.1%)	
Upper secondary education	57 (27.4%)	39 (29.8%)	18 (23.4%)	
Tertiary education or above	129 (62.0%)	78 (59.5%)	51 (66.2%)	
^1^ Family income, *N* (%)				0.937
Low income	68 (32.7%)	44 (33.6%)	24 (31.2%)	
Median income	36 (17.3%)	22 (16.8%)	14 (18.2%)	
High income	93 (44.7%)	59 (45.0%)	34 (44.2%)	
Infants’ vitamin D supplementation intake, *N* (%)				0.211
Yes	18 (8.7%)	14 (10.7%)	4 (5.2%)	
No	189 (90.9%)	117 (89.3%)	72 (93.5%)	
Having a multiparous mother				0.001
Yes	94 (45.2%)	48 (36.6%)	46 (59.7%)	
No	112 (53.8%)	82 (62.6%)	30 (39.0%)	
Breastfeeding, *N* (%)				<0.001
Yes	142 (68.3%)	72 (55.0%)	70 (90.9%)	
Partially	76 (36.5%)	53 (40.5%)	23 (29.9%)	
Exclusively	66 (31.7%)	19 (14.5%)	47 (61.0%)	
No	66 (31.7%)	59 (45.0%)	7 (9.1%)	
Use of sun cream, *N* (%)				0.128
Yes	42 (20.2%)	22 (16.8%)	20 (26.0%)	
No	151 (72.6%)	96 (73.3%)	55 (71.4%)	
Mothers with smoking history, *N* (%)				0.541
Yes	12 (5.8%)	9 (6.9%)	3 (3.9%)	
No	192 (92.3%)	120 (91.6%)	72 (93.5%)	

^1^ Family incomes based on median monthly household income for domestic households: low income = < HKD $29999; median income = $30000–$39999; high income = > $39999. Independent t-test for numeric data, Chi-square Test for categorical data.

**Table 2:** Factors affecting vitamin D insufficiency or deficiency risk among infants aged 2 to 6 months.

	Risk of Vitamin D Insufficiency (25(OH)D <50nmol/L)					
	OR (95% CI)	*p*	aOR (95% CI) ^+^	*p*	aOR (95% CI) ^$^	*p*
Being breastfed	9.10 (3.69, 22.48)	<0.001	9.80 (3.81, 25.21)	<0.001	12.81 (4.47, 36.71)	<0.001
Partially breastfed	4.12 (1.56, 10.91)	0.004	4.35 (1.57, 12.03)	0.005	5.62 (1.82, 17.33)	0.003
Exclusively breastfed	23.00 (8.49, 62.31)	<0.001	27.76 (9.47, 81.34)	<0.001	36.14 (10.84, 120.54)	<0.001
Other risk factors						
As a girl	1.71 (0.97, 3.02)	0.064	1.87 (1.02, 3.41)	0.041	4.30 (1.87, 9.91)	0.001
From a low-income family	0.92 (0.50, 1.70)	0.791	0.87 (0.45, 1.65)	0.662	1.13 (0.46, 2.79)	0.789
Having a multiparous mother	2.62 (1.46, 4.69)	0.001	3.32 (1.76, 6.29)	<0.001	3.74 (1.69, 8.25)	0.001
No vitamin supplementation intake	2.15 (0.68, 6.80)	0.191	2.12 (0.65, 6.93)	0.216	3.93 (0.92, 16.80)	0.065
Applied sun cream by parents	1.59 (0.80, 3.16)	0.190	1.96 (0.92, 4.17)	0.080	4.08 (1.29, 12.92)	0.017
Mothers with a smoking history	0.56 (0.15, 2.12)	0.390	0.35 (0.09, 1.46)	0.151	0.55 (0.09, 3.21)	0.504

+ Adjusted for age and gender of the child and the mean UV index at month of interview, $ Further adjusted mutually.

## Data Availability

The data that support the findings of this study are available from the corresponding author upon reasonable request.
